# Supplementary material for: Structures of Tetrahymena thermophila respiratory megacomplexes on the tubular mitochondrial cristae
Source: Nat Commun. 2023 May 29;14:2542. doi: 10.1038/s41467-023-38158-5 (PMC10227065; doi:10.1038/s41467-023-38158-5)
Supplement: Supplementary file 1 — Supplementary Information [file 41467_2023_38158_MOESM1_ESM.pdf]

Supplementary information

Structures of *Tetrahymena thermophila* Respiratory Megacomplexes on the Tubular Mitochondrial Cristae

Fangzhu Han[1,2]†, Yiqi Hu[1,2]†, Mengchen Wu[1,2], Zhaoxiang He[1,2], Hongtao Tian[1,2], Long Zhou[1,2]*

[1]Department of Biophysics, Zhejiang University School of Medicine; Hangzhou, Zhejiang Province, 310058, China
[2]Department of Critical Care Medicine of Sir Run Run Shaw Hospital, Zhejiang University School of Medicine; Hangzhou, Zhejiang Province, 310058, China
*Corresponding author. Email: longzhou@zju.edu.cn
†These authors contributed equally to this work

This file includes:

Supplementary Discussion
Supplementary Figs. 1 to 15
Supplementary Table 1
Supplementary References

Supplementary Discussion

Ciliate-specific subunits of Tt-CI

The highly divergent electron transport chain of *T. thermophila* was first described in the previous structural investigation of Tt-SC I+III$_2$ and Tt-CIV$_2$[1]. Out of the 21 ciliate-specific subunits of Tt-CI, three (NDUTT1, NDUTT2 and NDUTT15) are associated with Tt-PA and share structural homology with lipid-A-disaccharide synthases, acyl-CoA synthetase (AMP-forming)/AMP-acid ligase II (E.C. 6.2.1) NT domain and α-solenoid fold, respectively[1]. In Tt-CI MA, the NT globular domain of NDUTT6 and the CT helix of NDUTT12 that parallelly transverse the bottom of Tt-CI MA jointly form the IMS contact Site 3 between Tt-CI MA and Tt-CIV$_2$ in the megacomplex as above described (Fig. 2e). A cluster of TMHs from ciliate-specific subunits including NDUTT5, NDUTT8, NDUTT11, NDUTT16 and NDUTT17 interdigitate with the ND5a NT domain and reinforce the contact

Site 2 connecting Tt-CI MA, Tt-CIV$_2$ and Tt-CII (Fig. 2c, Supplementary Fig. 11g). NDUTT3 and NDUTT4 form the Tt-specific MA toe-bridge structure that constitutes an additional interaction site between Tt-CI and Tt-CIII$_2$[1], precludes canonical CIV association to CI MA toe but contact Tt-CIV$_2$ in the neighbouring megacomplex as revealed by the Tt-MC (IV$_2$+I+III$_2$+II)$_2$ structure (Supplementary Fig. 11h). Moreover, an additional cluster of ciliate-specific TM subunits including NDUTT7, NDUTT10 and NDUTT13 associate with Tt-CI MA heel in a position close to COXTT4 of Tt-CIV$_2$ and might represent an yet unknown contact site to neighbouring megacomplex structures (Supplementary Fig. 11i). Taken together, most of the ciliate-specific subunits in Tt-CI MA previously with unknown functions in Tt-SC I+III$_2$ are now demonstrated with structural roles in bridging to Tt-CIV$_2$ and Tt-CII and facilitating megacomplex formation in the present study.

A wedged Q is found between Tt-CIII$_2$ Q cavities

Apart from the *en route* Q sites, no Q density was found in the Tt-CIII$_2$ Q_p or Q_N cavities. Instead of two Q molecules harboured between the b_H hemes in the recently reported Tt-SC IV$_2$+I+III$_2$+II structure[2], a pair of cardiolipin molecules could be identified here in Tt-MC IV$_2$+(I+III$_2$+II)$_2$ by the characteristic densities of diphosphatidylglycerol with four alkyl tails (Supplementary Fig. 9g). Meanwhile in agreement with previous study of Tt-SC I+III$_2$[2], density resembling the single isoprenoid tail of Q is found at a non-canonical position between the two b_L hemes in both Tt-SC I+III$_2$[1] and Tt-MC IV$_2$+(I+III$_2$+II)$_2$ structures (Supplementary Fig. 9a). Missing of Q head density may suggest that it can adopt positions facing either b_L heme thus becomes averaged out during refinement. This is in contrast to mammalian CIII$_2$ which displays protein obstruction between the two Q cavities in the respirasome (Supplementary Fig. 9a, b)[3], therefore relying on a canonical Q_p-Q_N architecture to fulfil the Q cycle mechanism. The presence of Q in between b_L hemes has been observed for the actinobacterial SC III$_2$+IV$_2$[4] and implies additional electron transfer from or towards either b_L heme. Full exchange between QH_2 and Q accompanies movements of two protons across the IMM[5,6]. Spatial distance between the wedged Q and Rieske head of the same side precludes the scenario that such protons are carried to the IMS side via Rieske head movement when carrying electron over to c_1 heme[7]. Although nearby protonatable COB_Arg^{180} and continuous tunnels leading to both matrix and IMS sides are identified, lack of further protonatable residues lining the matrix tunnel and spatial blockage by a well resolved phosphatidylcholine in middle of the IMS tunnel make successful proton deliveries unlikely (Supplementary Fig. 9c). Therefore, instead of a Q redox site, this wedged Q more

likely serves as a single-electron buffer that keeps the b_L heme oxidized under high electron supply by turning into a semi-Q, as suggested for the actinobacterial SC III_2+IV_2[4]. Additionally, its position in middle of the two b_L hemes implicates a role as a single-electron relay station compensating the 14.5 Å distance beyond direct electron transfer between them[1]. In this way, it could regulate reductive stresses between the two Tt-$CIII_2$ electron pathways in Tt-MC $IV_2+(I+III_2+II)_2$ by coordinating in-coming electron supplies from Tt-CI and Tt-CII.

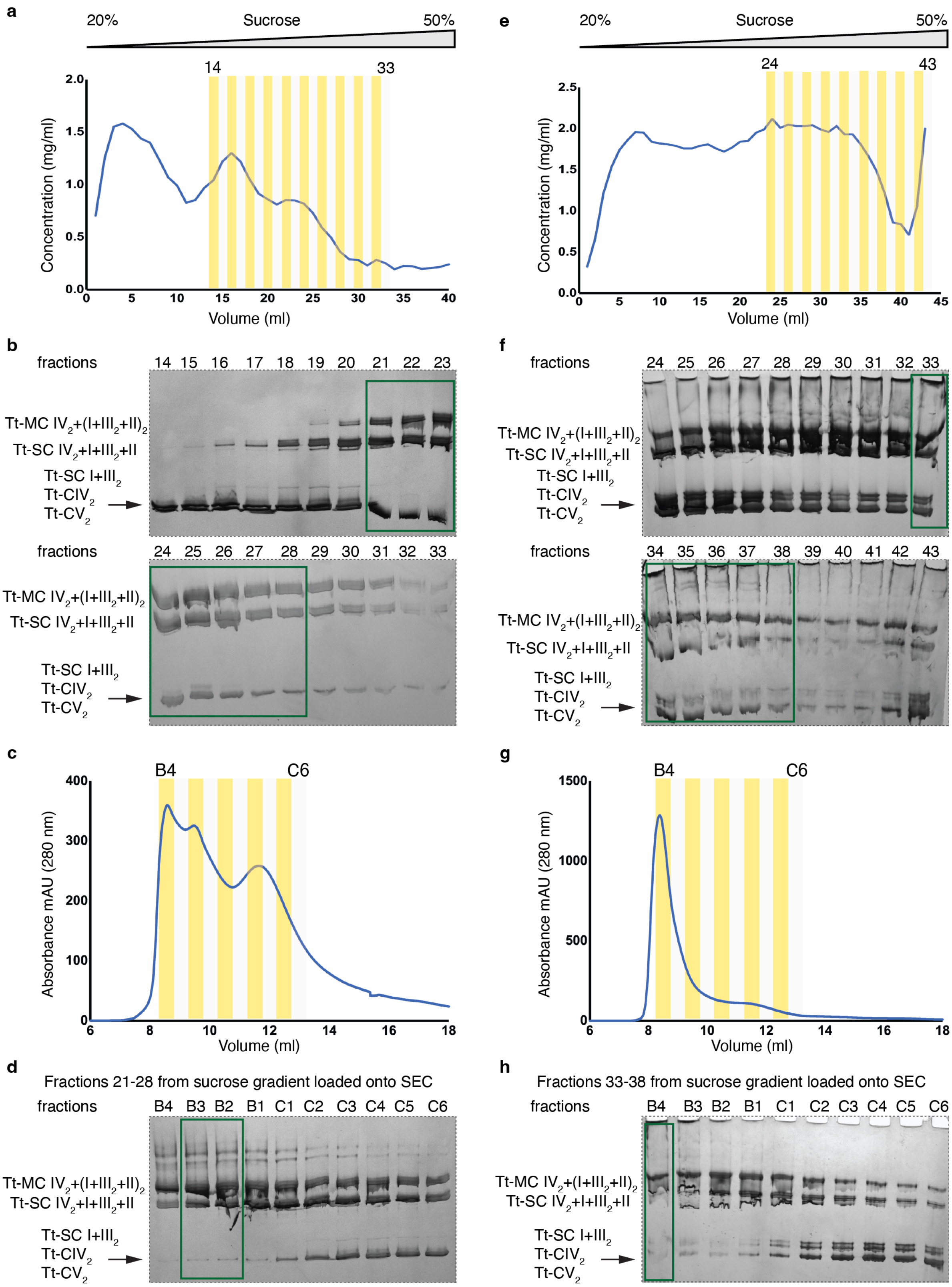

Supplementary Fig. 1. Purification of *T. thermophila*'s respiratory megacomplexes. (a and e) Chromatogram of sucrose gradient fractionation of LMNG **(a)** and digitonin **(e)**-extracted *T. thermophila* mitochondrial membrane megacomplexes. Yellow vertical stripes represent fractions shown in (**b**) and **(f)**. (**b and f**) BN-PAGE of fractions from **(a)** and **(e)**, fractions pooled and loaded onto SEC chromatography are highlighted by green boxes. (**c and g**) Superose 6 Increase 10/300 GL column (GE Healthcare) chromatogram of indicated

pooled fractions. **(d and h)** BN-PAGE of fractions from **(c)** and **(g)**, fraction(s) used for cryo-EM grid preparations are highlighted by green boxes. Megacomplex purification from LMNG **(a-d)** and digitonin **(e-h)** solubilized mitochondrial membrane are independently repeated for seven and five times with similar results respectively. Source data are provided as a Source Data file.

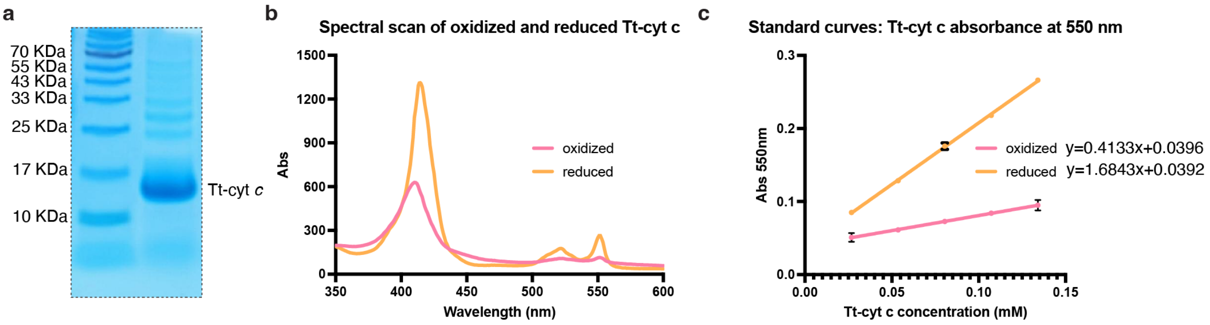

Supplementary Fig. 2. Recombinant expression and extinction coefficient measurement of Tt-cyt *c*. (a) SDS-PAGE of recombinantly expressed Tt-cyt *c* stained by Coomassie brilliant blue. **(b)** Spectral scan of recombinantly expressed Tt-cyt *c*, reduced and oxidized by DDT and H_2O_2 (v/v) respectively. Not the peak at 550 nm is present for reduced but not oxidized Tt-cyt *c*. **(c)** Standard curves for reduced and oxidized Tt-cyt *c*, absorbance at 550 nm against cyt *c* concentration in mM. Values are averages of three technical measurements from the same sample ±SEM. Equations are listed for linear regression done for the means. Source data are provided as a Source Data file.

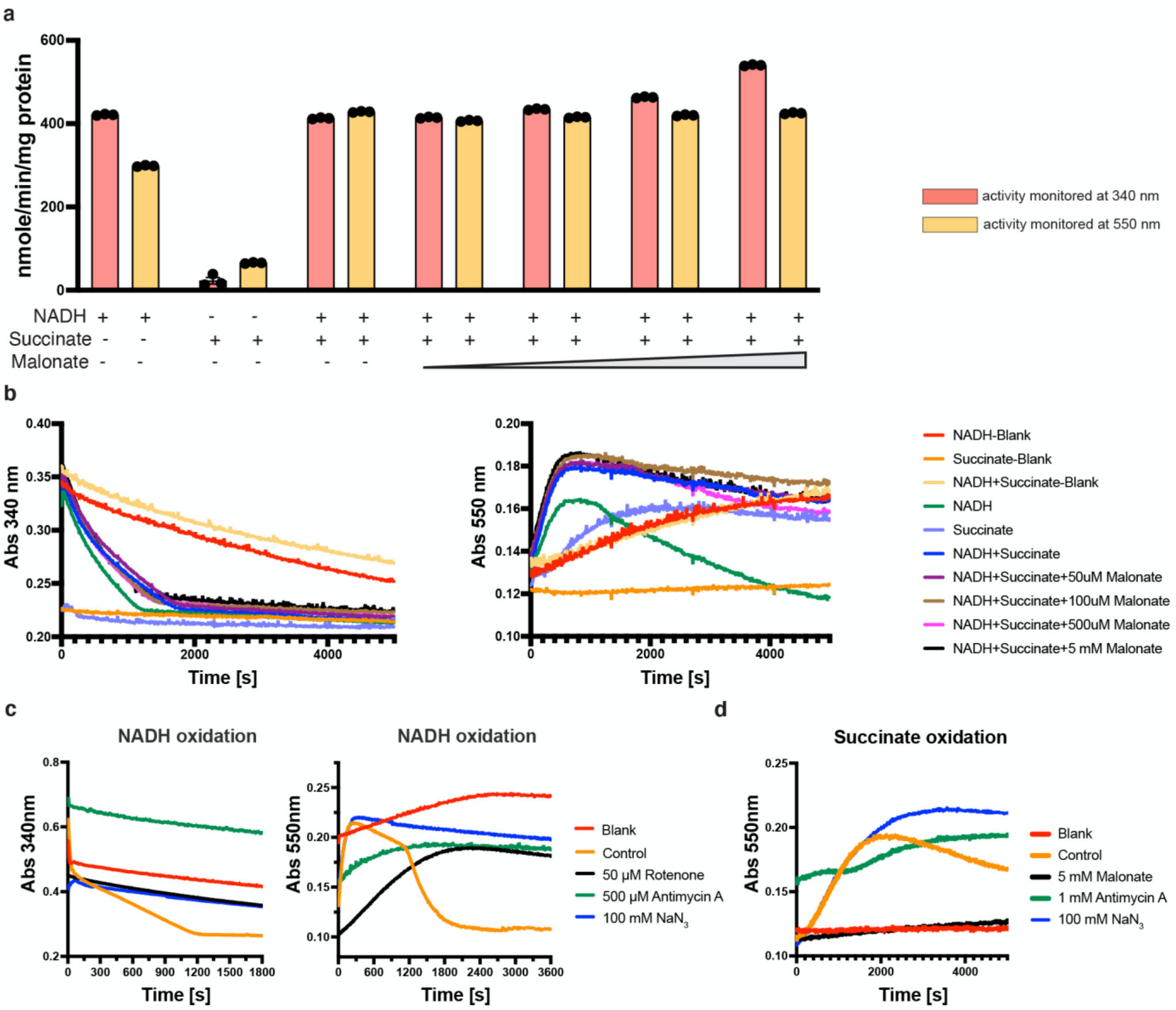

Supplementary Fig. 3. Kinetic measurements of NADH:O_2 and succinate:O_2 oxidoreductase activities of Tt-MC IV_2+(I+III_2+II)$_2$. (a) Comparison of the activities of individual and simultaneous NADH and succinate oxidation by purified Tt-MC IV_2+(I+III_2+II)$_2$, in the absence or presence of 50 µM, 100 µM, 500 µM or 5 mM Tt-CII specific inhibitor malonate. Activities are monitored at 340 nm by oxidation of NADH (red bars) or at 550 nm by reduction of cyt *c* (yellow bars). Values are averages of three technical measurements from a single purified sample ±SEM. **(b-d)** Kinetic traces of measurements in panel **(a)** of this figure **(b)**, panel **(d-f)** of Fig. 1 **(c)** and panel **(g-h)** of Fig. 1 **(d)**. 2 nM and 10 nM Tt-megacomplex samples are used for **(a, b)** and **(c, d)**, respectively. Different reaction conditions are color-coded and when there are two sub-panels present, they represent absorbance monitored at 340 nm (left) and 550 nm (right) of the same reaction. Note the NADH oxidation and cyt *c* reduction rates are calculated by slopes of the initial linear phases in kinetic traces monitored at 340 nm and 550 nm respectively. Cyt *c* oxidation rates are calculated by slopes of linear regions in the last decline of 550 nm absorbance (see **Fig. 1 d-h**and **Methods**). Values are averages of three technical measurements from a single purified sample, error bars representing SEM are not shown for clarity. Source data are provided as a Source Data file.

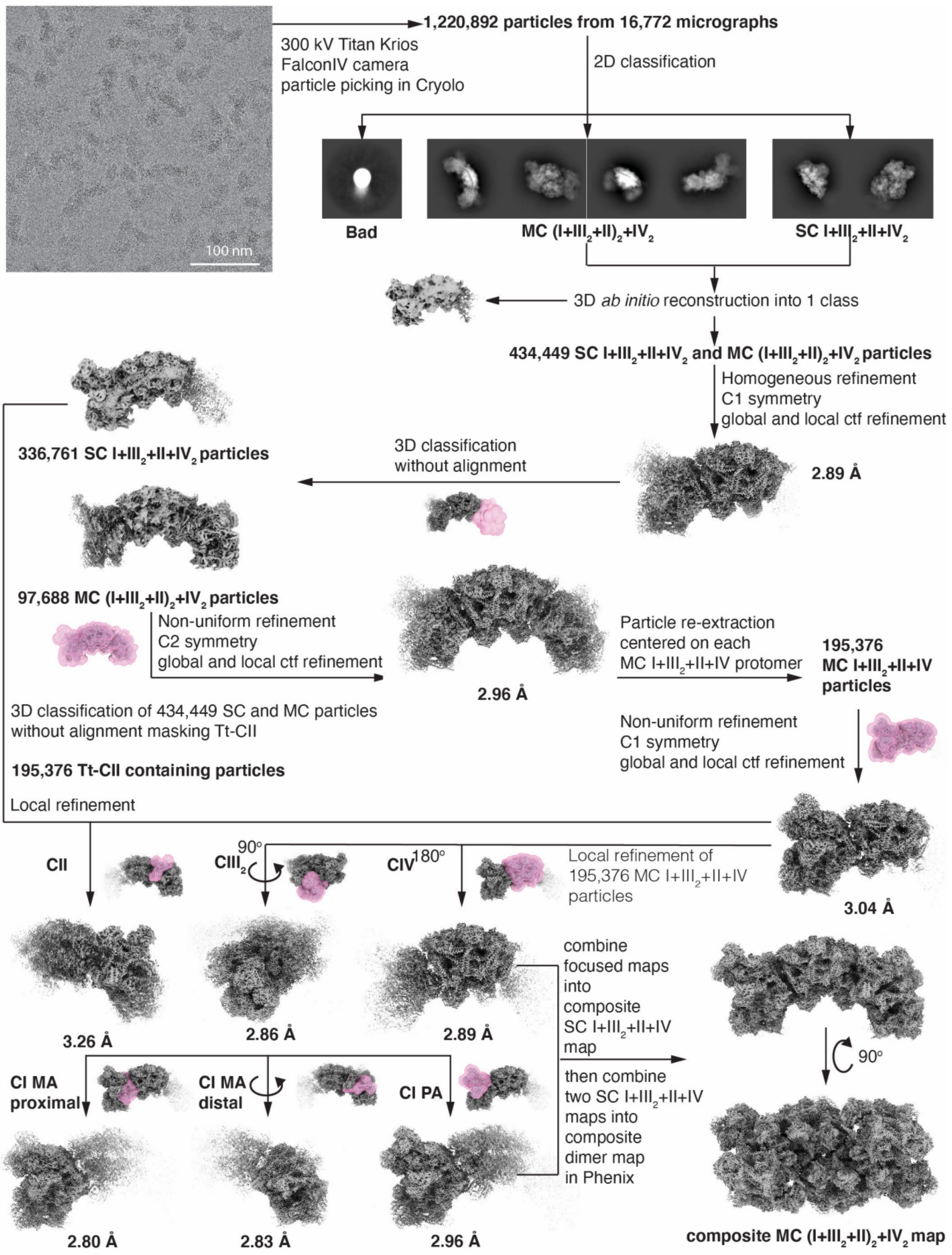

Supplementary Fig. 4. Cryo-EM image processing of Tt-MC IV$_2$+(I+III$_2$+II)$_2$. A total of 16,772 images were collected from a 300 kV Titan Krios microscope with Falcon IV camera, from which 1,220,892 particles were initially picked using crYOLO. In cryoSPARC, a total of 434,449 Tt-SC IV$_2$+I+III$_2$+II and Tt-MC IV$_2$+(I+III$_2$+II)$_2$ particles were obtained after 2D classification and refined to an overall resolution of 2.89 Å. Focused 3D classification separated 336,761 Tt-SC IV$_2$+I+III$_2$+II particles from 97,688 Tt-MC IV$_2$+(I+III$_2$+II)$_2$ particles. After C2 symmetry-expansion and re-extraction, local refinements of Tt-CI peripheral arm, proximal portion of Tt-CI membrane arm, distal portion of Tt-CI membrane arm, Tt-CII$_2$,Tt-CIII$_2$ and Tt-CIV achieved resolutions from 2.80-3.26 Å, from which a

composite Tt-MC IV_2+(I+III_2+II)$_2$ map was generated in Phenix. Masks used for individual local refinements are indicated as pink transparent surfaces.

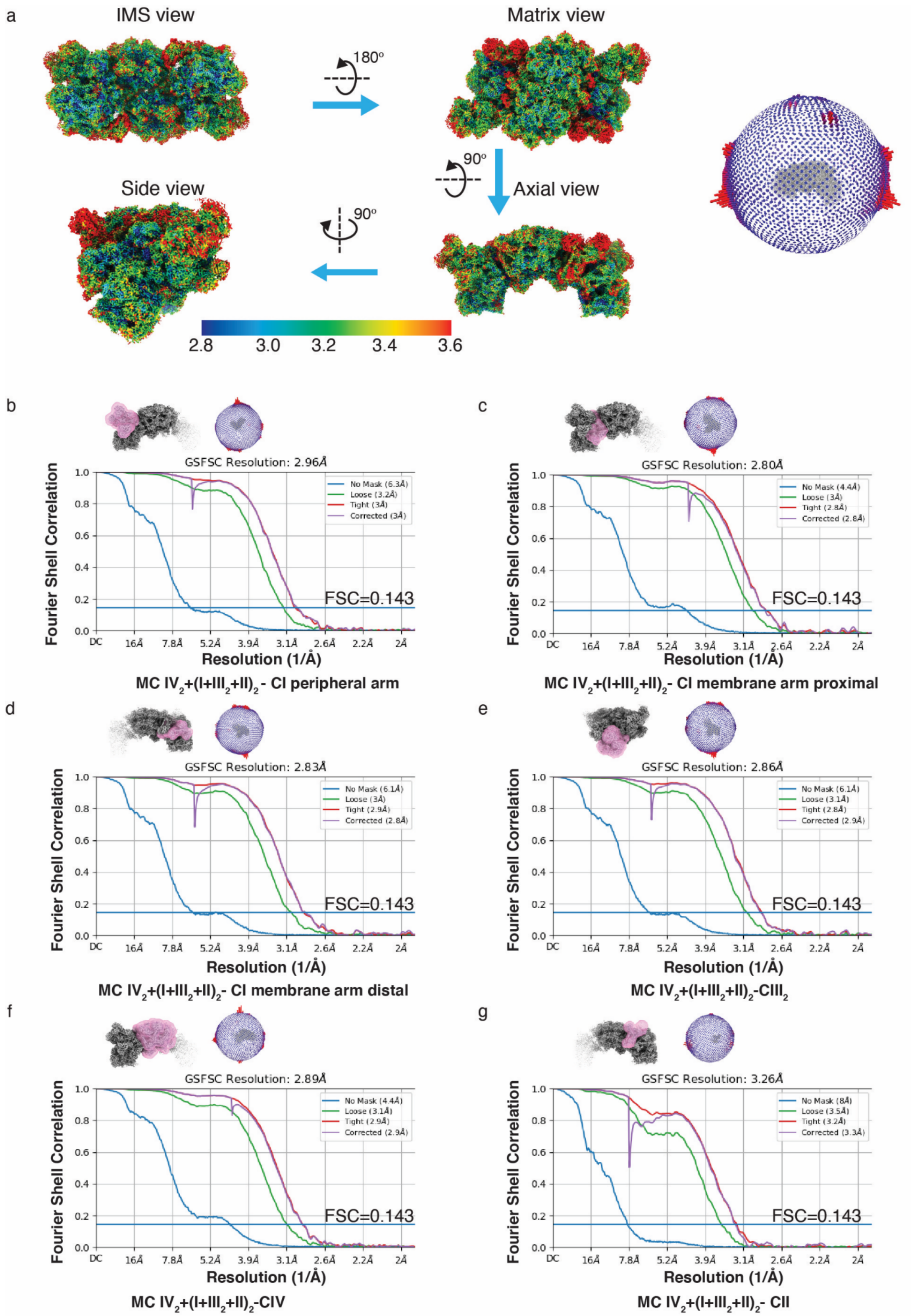

Supplementary Fig. 5. Local resolution maps and Fourier shell correlation (FSC) curves of Tt-MC IV$_2$+(I+III$_2$+II)$_2$. (a) Local resolution plotted on composite maps and presented

by different views. **(bto g)** FSC curves (gold standard FSC=0.143 for resolution estimation) of Tt-MC IV_2+(I+III_2+II)$_2$.

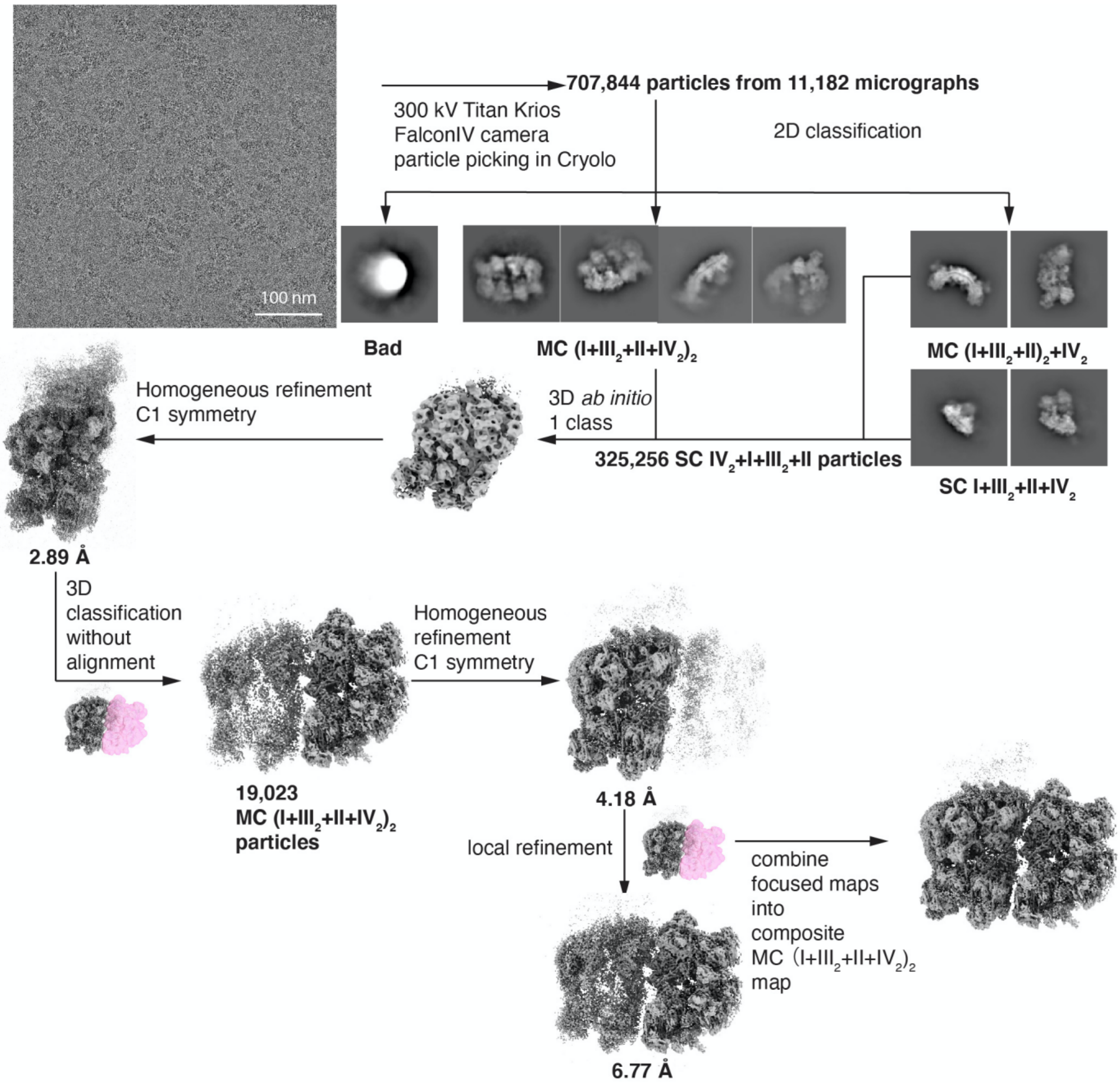

Supplementary Fig. 6. Cryo-EM image processing of Tt-MC (IV$_2$+I+III$_2$+II)$_2$. A total of 11,182 images were collected from a 300 kV Titan Krios microscope with Falcon IV camera, from which 707,844 particles were initially picked using crYOLO. In cryoSPARC, a total of 325,256 particles containing Tt-SC IV$_2$+I+III$_2$+II were obtained after 2D classification and refined to an overall resolution of 2.89 Å. Focused 3D classification obtained 19,023 Tt-MC (IV$_2$+I+III$_2$+II)$_2$ particles, overall refinement of which gave a resolution of 4.18 Å. Local refinements of the less-clear part of IV$_2$+I+III$_2$+II gave a resolutions from 6.77 Å. The two maps were used to form a composite Tt-MC IV$_2$+(I+III$_2$+II)$_2$ map in Phenix. Masks used for individual local refinements are indicated as pink transparent surfaces.

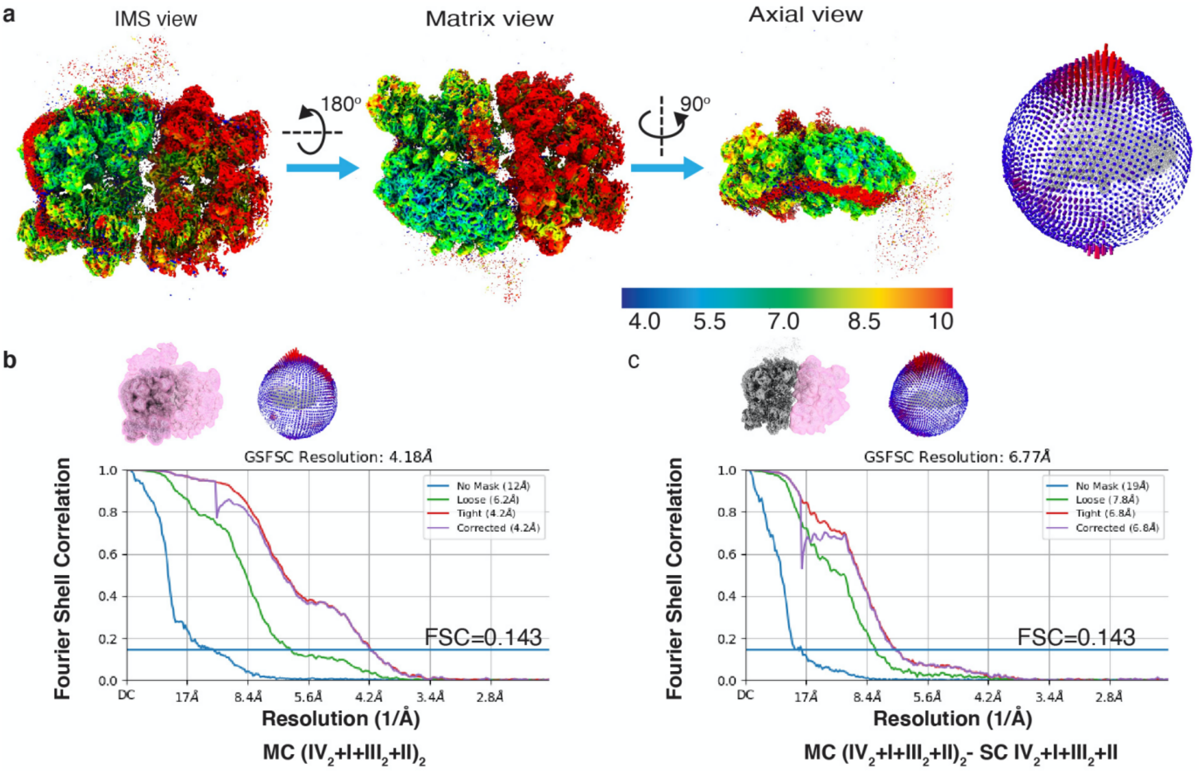

Supplementary Fig. 7. Local resolution maps and Fourier shell correlation (FSC) curves of Tt-MC (IV_2+I+III_2+II)$_2$. (a) Local resolution plotted on composite maps and presented by different views. **(bto c)** FSC curves (gold standard FSC=0.143 for resolution estimation) of Tt-MC (IV_2+I+III_2+II)$_2$.

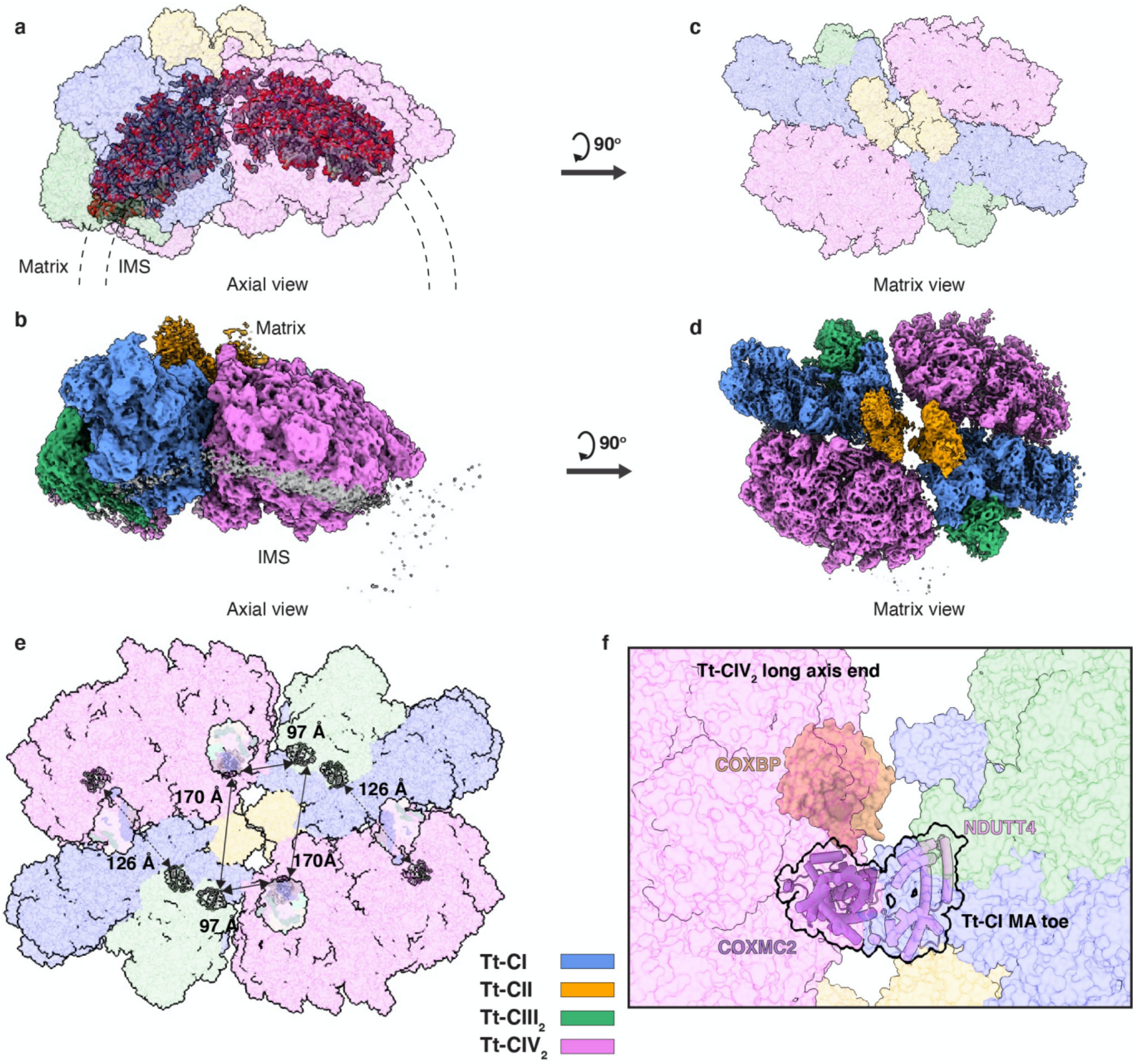

Supplementary Fig. 8. Structure of Tt-MC (IV$_2$+I+III$_2$+II)$_2$. (a) Axial and **(c)** matrix side views of Tt-MC (IV$_2$+I+III$_2$+II)$_2$ with individual ETC complexes are shown in transparent surfaces and coloured as indicated. In **(a)** cross-section of the ciliate tubular cristae is overlaid with the structure. Phospholipids are shown as spheres and indicate the curvature of the megacomplex membrane domain. **(b)** Axial and **(d)** matrix side views of Tt-MC (IV$_2$+I+III$_2$+II)$_2$ composite map coloured as in **(a)**. **(e)**The theoretical cyt *c* binding sites on Tt-CIII$_2$ and Tt-CIV$_2$ are obtained by alignment with cyt *c*-bound mammalian Tt-CIII$_2$ (PDB: 5IY5)[8] and Tt-CIV$_2$ (PDB: 3CX5)[9] structures and indicated by cartoon representations of cyt *c*. Solid and dashed double arrows indicate kinetically favourable and unfavourable cyt *c* diffusion distances. **(f)** Zoom-in of the interaction site between Tt-CI MA toe and Tt-CIV$_2$ in Tt-MC (IV$_2$+I+III$_2$+II)$_2$. Key subunits are shown as cylindrical cartoon, subunit COXBP representing the end of Tt-CIV$_2$'s long axis is shown as solid surface.

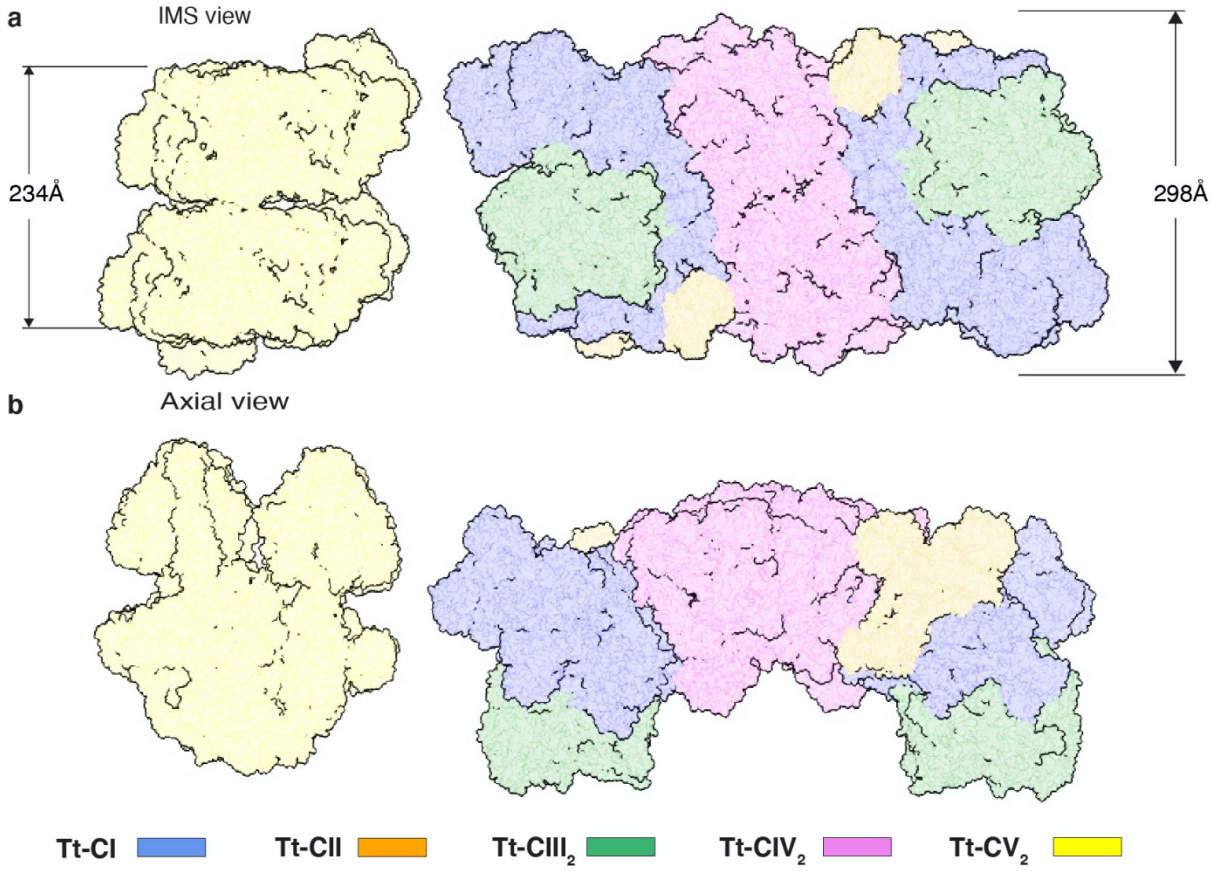

Supplementary Fig. 9. Comparisons of Tt-CV_4 and Tt-MC IV_2+(I+III2+II)$_2$. (a) IMS and **(b)** axial views of Tt-CV_4 and Tt-MC IV_2+(I+III_2+II)$_2$ are shown as coloured transparent surfaces. Their axial thicknesses of respective membrane domains are labelled.

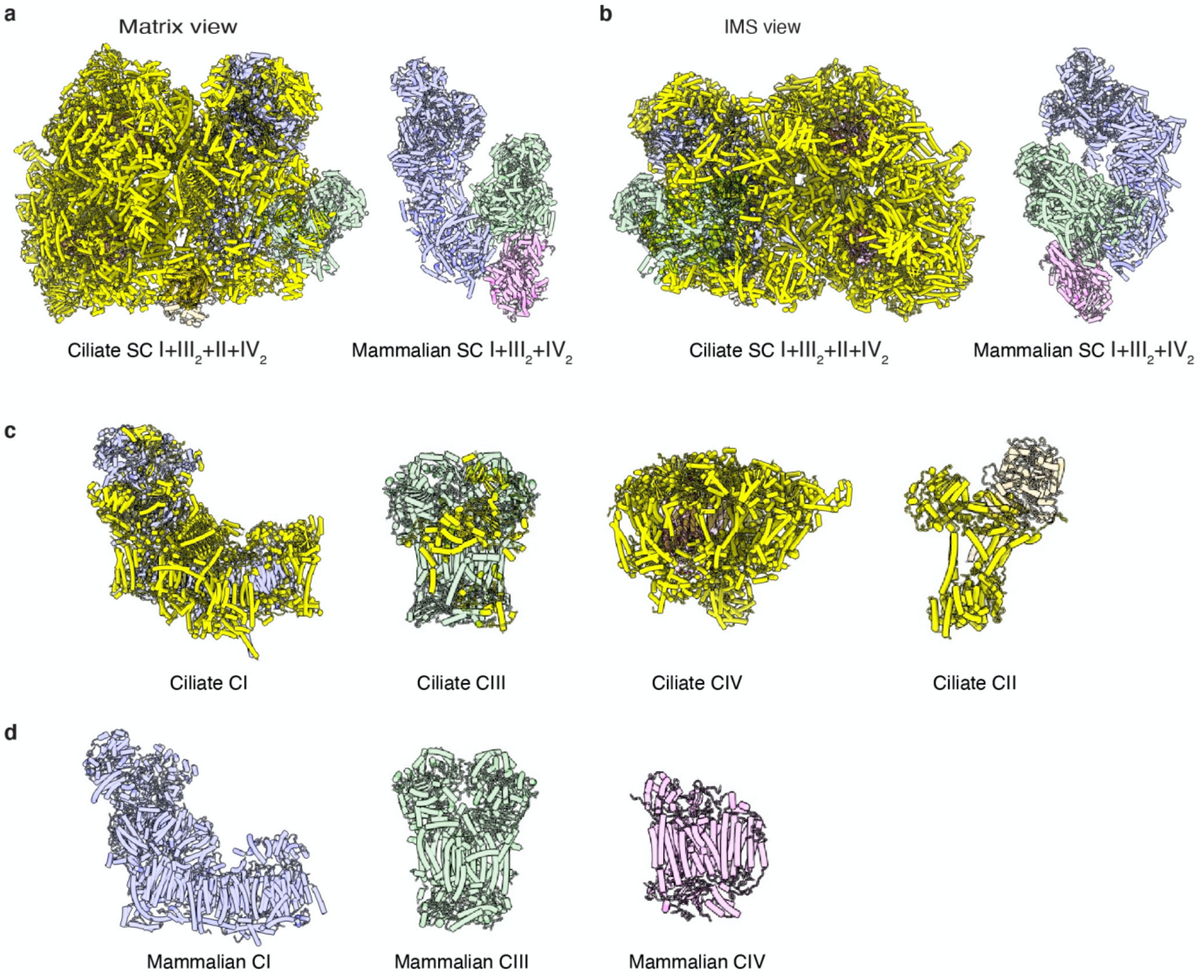

Supplementary Fig. 10. Comparisons of Tt-SC IV$_2$+I+III$_2$+II and mammalian respirasome. (a) Matrix and **(b)** IMS views of Tt-SC IV$_2$+I+III$_2$+II and mammalian respirasome (PDB 5J4Z)[3] are shown as cylindrical cartoon in context of transparent surfaces coloured by individual complexes, with conserved subunits coloured as in Fig. 1 and ciliate-specific subunits and extensions coloured yellow. The individual complexes of Tt-SC IV$_2$+I+III$_2$+II **(c)** and mammalian respirasome **(d).**

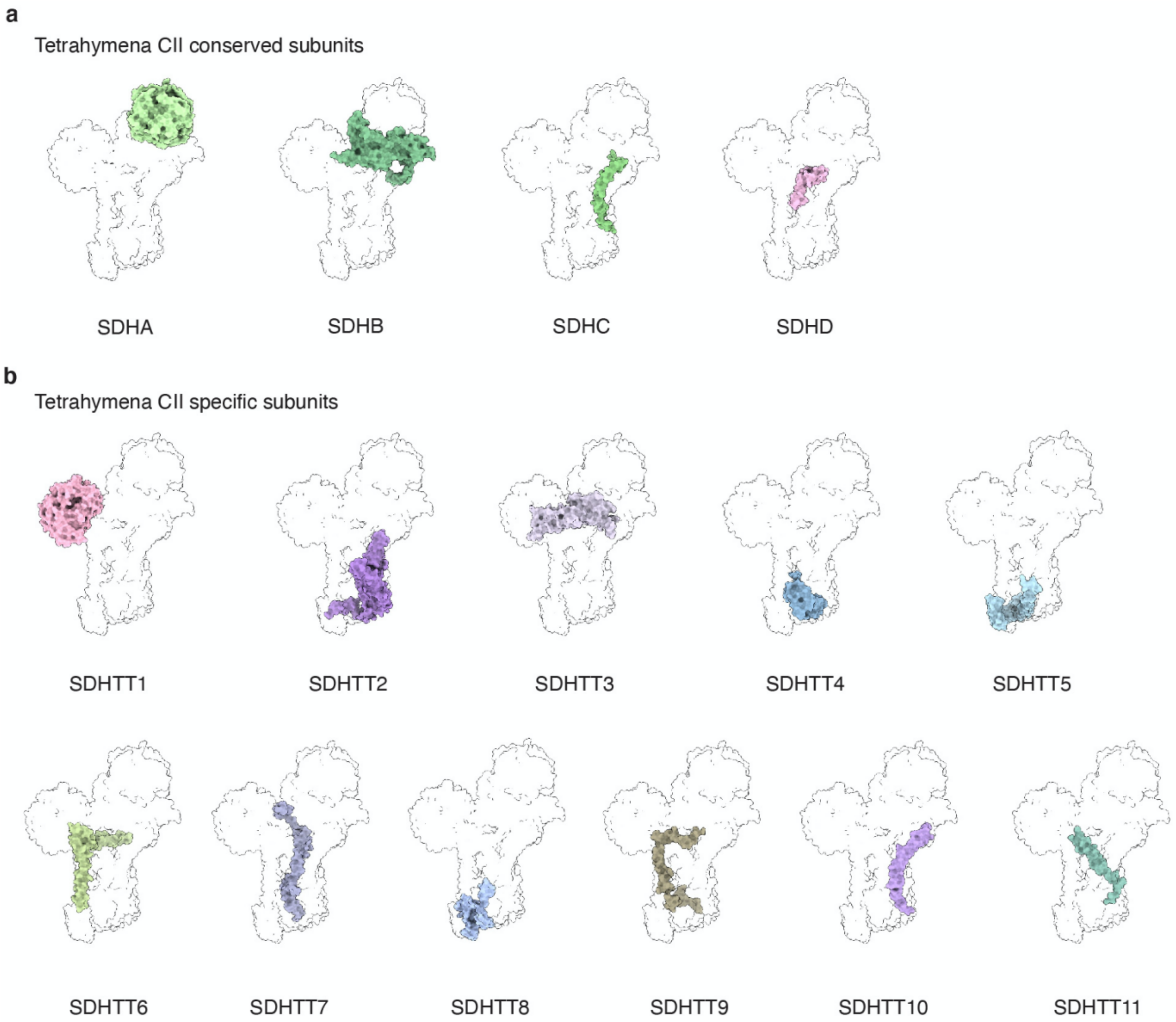

Supplementary Fig. 11. The structure and spatial organization of Tt-CII subunits. Opisthokonta-conserved **(a)** and ciliate-specific **(b)** Tt-CII subunits are shown as coloured solid surfaces in the context of the whole complex silhouette.

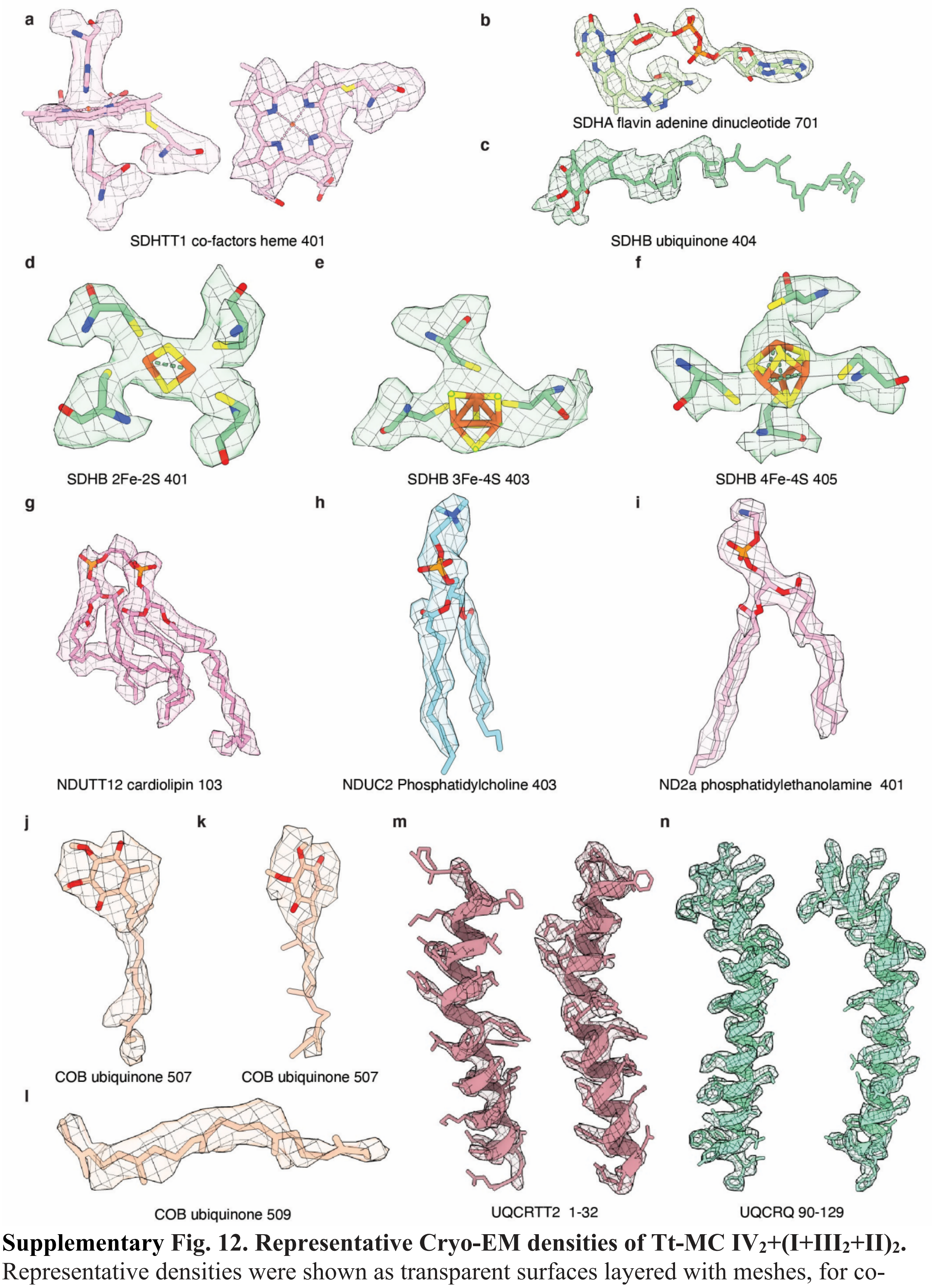

Supplementary Fig. 12. Representative Cryo-EM densities of Tt-MC IV$_2$+(I+III$_2$+II)$_2$. Representative densities were shown as transparent surfaces layered with meshes, for co-factors heme c **(a)**, flavin adenine dinucleotide (FAD) **(b)**, ubiquinone **(c)**, 2Fe-2S cluster **(d)**, 3Fe-4S cluster **(e)** and 4Fe-4S cluster **(f)** of Tt-CII; lipids including cardiolipin **(g)**, phosphatidylcholine **(h)** and phosphatidylethanolamine **(i)**; ubiquinones **(jto l)** and transmembrane helices of UQCRTT2 **(m)** and UQCRQ **(n)** of Tt-CIII$_2$.

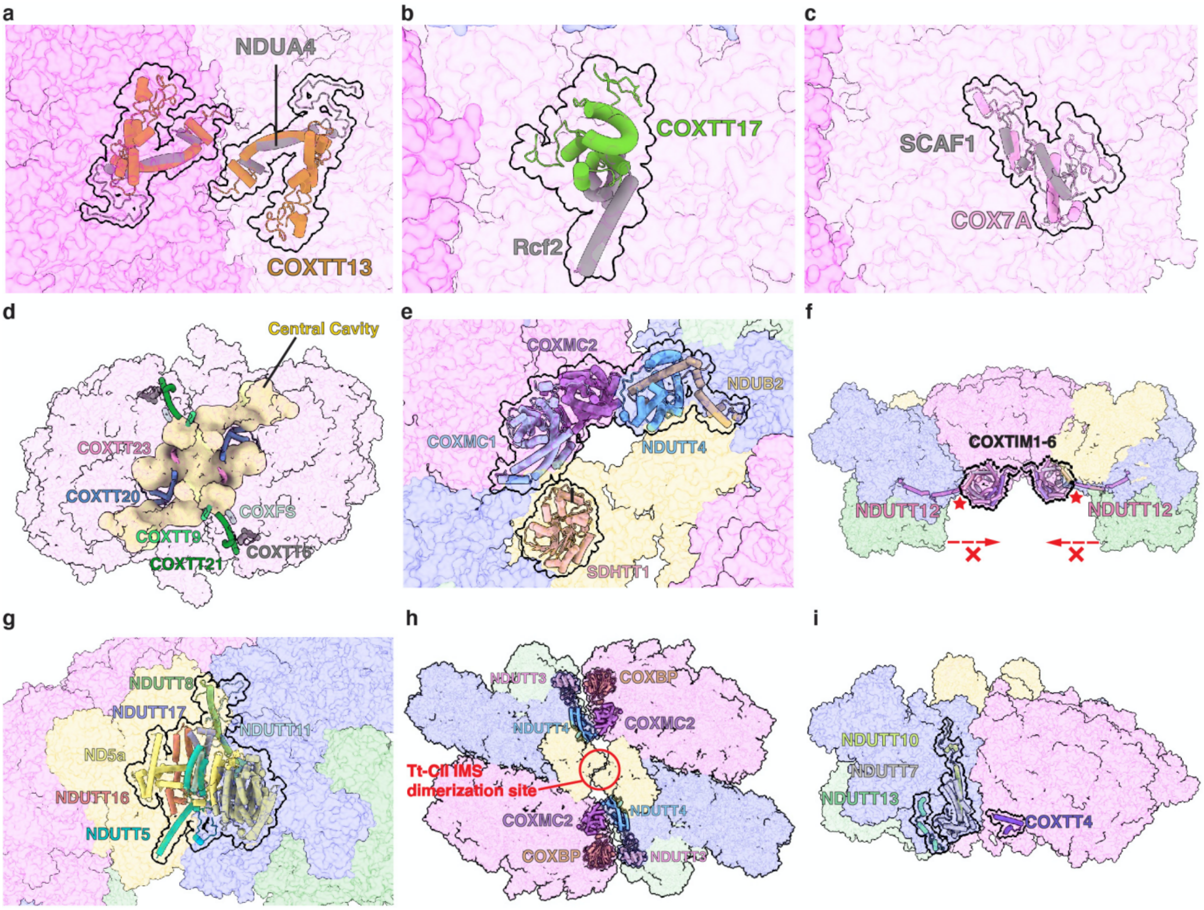

Supplementary Figure 13. Ciliate specific subunits of Tt-CIV2 contributes to megacomplex formation. Superpositions of assembly factors NDUA4 **(a)**, Rcf2 **(b)** and SCAF1 **(c)** to Tt-CIV$_2$, aligned by CIV core subunits COX1-3. Tt-CIV$_2$ is shown as transparent surface, with different shades of magenta indicating its two protomers. Opisthokont assembly factors and their structural homologues in Tt-CIV$_2$ are shown as cylindrical cartoons, coloured grey and by subunits as in Fig. 1c, respectively. **(d)** TMHs of Tt-CIV$_2$ subunits lining its central cavity at the dimer interface. **(e)** Mitochondrial carrier heterodimer COXMC1-2 interacts with SDHTT1 of Tt-CII, as well as NDUB2 and NDUTT4 of the neighbouring megacomplex. **(f)** Potential steric clashes, marked by red star, between NDUTT12 and COXTIM1-6, help to keep the two Tt-SC I+III$_2$ copies from coming closer into the cristae center, as marked by dashed arrows and red crosses. Ciliate specific subunits of Tt-CI and Tt-CIV$_2$ at interaction site 2 **(g)**, at Tt-CI MA toe **(h)** and at Tt-CI MA heel **(i)** respectively.

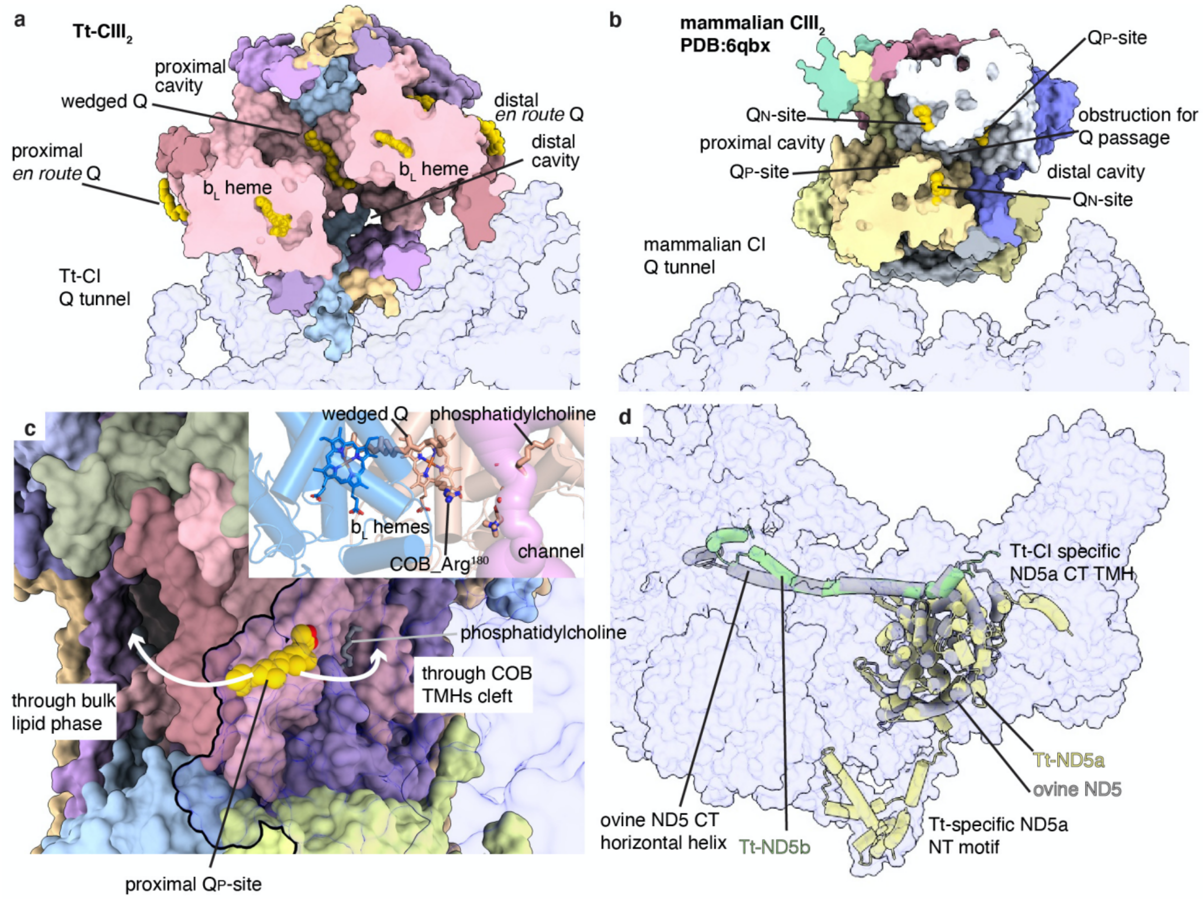

Supplementary Fig. 14. Comparison of Tt-$CIII_2$ and mammalian $CIII_2$. $CIII_2$ Q cavities of **(a)** Tt- and **(b)** mammalian (PDB: 6QBX)[10] SC I+III_2 are shown in the same view with equal clipping. CI and $CIII_2$ are shown as transparent and solid surfaces respectively, ubiquinones are shown as yellow atomic spheres. **(c)** Possible paths for QH_2 diffusion from Tt-CI Q tunnel into the Tt-$CIII_2$ proximal cavity. A phosphatidylcholine molecule in-between subunit COB TMHs is shown as atomic stick representing a hydrophobic cleft for QH_2 diffusion. **(Insert)** The wedged Q, shown in stick, and its nearby channel coloured in magenta. The nearby COB_Arg^{180} and a phosphatidylcholine molecule intercepting the detected channel are also shown in sticks. **(d)** Comparison of Tt-CI's subunits ND5a and ND5b (yellow) with *Ovis aries* CI subunit ND5 (grey) (PDB: 6QBX)[10].

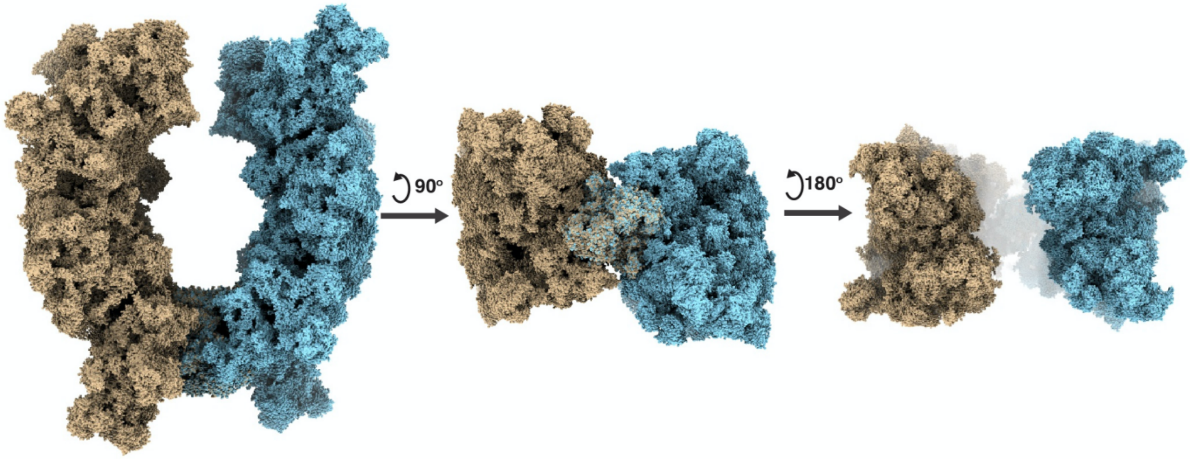

Supplementary Fig. 15. Hypothetical respiratory ring organized by aligning two Tt-MC $IV_2+(I+III_2+II)_2$. Hypothetical respiratory ring organized by aligning two Tt-MC $IV_2+(I+III_2+II)_2$ (shown as atoms coloured in sand and cyan) by the terminal Tt-$CIII_2$.

Supplementary Table 1: Cryo-EM data collection, refinement and validation statistics

	Dataset 1<br>Tt-MC IV_2+(I+III_2+II)$_2$<br>(EMDB-34373)<br>(PDB 8GYM)	Dataset 2<br>Tt-MC (IV_2+I+III_2+II)$_2$<br>(EMDB-34403)<br>(PDB 8GZU)
**Data collection and processing**		
	**Dataset 1**	**Dataset 2**
Magnification	140,000	105,000
Voltage (kV)	300	300
Electron exposure (e–/Å^2)	61.5	51.4
Defocus range (μm)	-0.8 to -2.0	-0.8 to -2.0
Pixel size (Å)	0.93	1.20
Symmetry imposed		
Initial particle images (no.)	16,772	11182
Final particle images (no.)		
Map resolution (Å)		
FSC threshold	CIV: 2.89<br>CI PA: 2.96<br>CI MAp: 2.80<br>CI MAd: 2.83<br>$CIII_2$: 2.86<br>CII: 3.26	IV_2+I+III_2+II: 4.18 and 6.77
Map resolution range (Å)	2.80-3.26	4.18-6.77
**Refinement**		
Initial model used (PDB code)	7tgh,7w5z	7tgh,7w5z
Model resolution (Å)	3.14	
FSC threshold	0.5	0.5
Model resolution range (Å)		
Map sharpening *B* factor (Å^2)	CIV: 86.9<br>CI PA: 94.9<br>CI MAp: 86.5<br>CI MAd: 89.5<br>$CIII_2$: 93.5<br>CII: 93.0	IV_2+I+III_2+II: 34.9 and 269.6
Model composition		
Non-hydrogen atoms	570,294	448,387
Protein residues	66,356	90,071
Ligands	512	116
B factors (Å^2)		
Protein	52.85	53.33
Ligand	18.25	45.29
R.m.s. deviations		
Bond lengths (Å)	0.007	0.004
Bond angles (°)	0.64	1.101
Validation		
MolProbity score	1.77	1.04
Clashscore	9.57	1.17
Poor rotamers (%)	1.07	0.00
Ramachandran plot		
Favored (%)	96.37	96.71
Allowed (%)	3.62	3.27
Disallowed (%)	0.01	0.02

Supplementary References

1. Zhou, L., Maldonado, M., Padavannil, A., Guo, F. & Letts, J. A. Structures of Tetrahymena’s respiratory chain reveal the diversity of eukaryotic core metabolism. *Science (New York, N.Y.)* **376**, 831–839 (2022).
2. Mühleip, A., Flygaard, R. K., Baradaran, R. & Haapanen, O. Structural basis of mitochondrial membrane bending by the I – II – III 2 – IV 2 supercomplex. (2023). doi:10.1038/s41586-023-05817-y
3. Letts, J. A., Fiedorczuk, K. & Sazanov, L. A. The architecture of respiratory supercomplexes. *Nature* **537**, 644–648 (2016).
4. Kao, W. *u.c.* Structural basis for safe and efficient energy conversion in a respiratory supercomplex. *Nature communications* **13**, 545 (2022).
5. Sarewicz, M. *u.c.* Catalytic reactions and energy conservation in the cytochrome bc1 and b6f complexes of energy-transducing membranes. *Chemical Reviews* **121**, 2020–2108 (2021).
6. Letts, J. A. & Sazanov, L. A. Clarifying the supercomplex: the higher-order organization of the mitochondrial electron transport chain. *Nature structural & molecular biology* **24**, 800–808 (2017).
7. Link, T. A. The role of the "Rieske" iron sulfur protein in the hydroquinone oxidation (Q(P)) site of the cytochrome bc1 complex. The "proton-gated affinity change" mechanism. *FEBS letters* **412**, 257–64 (1997).
8. Shimada, S. *u.c.* Complex structure of cytochrome c-cytochrome c oxidase reveals a novel protein-protein interaction mode. *The EMBO journal* **36**, 291–300 (2017).
9. Solmaz, S. R. N. & Hunte, C. Structure of complex III with bound cytochrome c in reduced state and definition of a minimal core interface for electron transfer. *The Journal of biological chemistry* **283**, 17542–9 (2008).
10. Letts, J. A., Fiedorczuk, K., Degliesposti, G., Skehel, M. & Sazanov, L. A. Structures of Respiratory Supercomplex I+III2 Reveal Functional and Conformational Crosstalk. *Molecular cell* **75**, 1131-1146.e6 (2019).
