# Supplementary material for: Structures of Tetrahymena thermophila respiratory megacomplexes on the tubular mitochondrial cristae
Source: Nat Commun. 2023 May 29;14:2542. doi: 10.1038/s41467-023-38158-5 (PMC10227065; doi:10.1038/s41467-023-38158-5)
Supplement: Supplementary file 3 — Description of Additional Supplementary Files [file 41467_2023_38158_MOESM3_ESM.pdf]

File Name: Supplementary Data 1

Description: Tt-MC IV2+(I+III2+II)2 model summary

File Name: Supplementary Movie 1

Description: 360° rotation of Tt-MC IV_2+(I+III_2+II)$_2$ map and model, first as maps colored by different ETC complex, then as maps colored by different subunits, lastly as models colored by different subunits.

File Name: Supplementary Movie 2

Description: 360° rotation of Tt-MC (IV_2+I+III_2+II)$_2$ map and model, first as maps colored by different ETC complex, then as maps colored by different subunits, lastly as models colored by different subunits.
