# Supplementary material for: Structures of Tetrahymena thermophila respiratory megacomplexes on the tubular mitochondrial cristae
Source: Nat Commun. 2023 May 29;14:2542. doi: 10.1038/s41467-023-38158-5 (PMC10227065; doi:10.1038/s41467-023-38158-5)
Supplement: Supplementary file 7 — Reporting Summary [file 41467_2023_38158_MOESM7_ESM.pdf]

nature portfolio

Corresponding author(s): Long Zhou

Last updated by author(s): Apr 5, 2023

Reporting Summary

Nature Portfolio wishes to improve the reproducibility of the work that we publish. This form provides structure for consistency and transparency in reporting. For further information on Nature Portfolio policies, see our Editorial Policies and the Editorial Policy Checklist.

Statistics

For all statistical analyses, confirm that the following items are present in the figure legend, table legend, main text, or Methods section.

n/a	Confirmed	
☐	☒	The exact sample size (*n*) for each experimental group/condition, given as a discrete number and unit of measurement
☐	☒	A statement on whether measurements were taken from distinct samples or whether the same sample was measured repeatedly
☒	☐	The statistical test(s) used AND whether they are one- or two-sided<br>*Only common tests should be described solely by name; describe more complex techniques in the Methods section.*
☒	☐	A description of all covariates tested
☒	☐	A description of any assumptions or corrections, such as tests of normality and adjustment for multiple comparisons
☐	☒	A full description of the statistical parameters including central tendency (e.g. means) or other basic estimates (e.g. regression coefficient) AND variation (e.g. standard deviation) or associated estimates of uncertainty (e.g. confidence intervals)
☒	☐	For null hypothesis testing, the test statistic (e.g. *F*, *t*, *r*) with confidence intervals, effect sizes, degrees of freedom and *P* value noted<br>*Give P values as exact values whenever suitable.*
☒	☐	For Bayesian analysis, information on the choice of priors and Markov chain Monte Carlo settings
☒	☐	For hierarchical and complex designs, identification of the appropriate level for tests and full reporting of outcomes
☒	☐	Estimates of effect sizes (e.g. Cohen's *d*, Pearson's *r*), indicating how they were calculated

Our web collection on statistics for biologists contains articles on many of the points above.

Software and code

Policy information about availability of computer code

Data collection	EPU 2
Data analysis	ChimeraX 1.4, Coot 0.9.6, CryoSPRAC 3.3.2, Phenix 1.20.1, Relion 4.0, CTFFIND4.1, crYOLO1.6.0, PyMOL 2.5.2, Prism9.3.1, BLAST web server, Phyre2 web server, PredictProtein web server

For manuscripts utilizing custom algorithms or software that are central to the research but not yet described in published literature, software must be made available to editors and reviewers. We strongly encourage code deposition in a community repository (e.g. GitHub). See the Nature Portfolio guidelines for submitting code & software for further information.

Data

Policy information about availability of data

All manuscripts must include a data availability statement. This statement should provide the following information, where applicable:

- Accession codes, unique identifiers, or web links for publicly available datasets
- A description of any restrictions on data availability
- For clinical datasets or third party data, please ensure that the statement adheres to our policy

The structural models of Tt-MC IV2+(I+III2+II)2 and Tt-MC (IV2+I+III2+CII)2 generated in this study have been deposited in the Protein Data Bank (PDB) under accession codes 8GYM [https://www.rcsb.org/structure/unreleased/8GYM] and 8GZU [https://www.rcsb.org/structure/unreleased/8GZU] respectively. The composite Cryo-EM maps of Tt-MC IV2+(I+III2+II)2 and Tt-MC (IV2+I+III2+CII)2 generated in this study have been deposited in the Electron Microscopy Database

(EMDB) under accession codes EMD-34373 and EMD-34403. Local refinements of CI peripheral arm, CI membrane arm distal region, CI membrane arm proximal region, CIII2, CIV and CII of Tt-MC IV2+(I+III2+II)2 generated in this study have been deposited in the EMDB under accession codes EMD-34380, EMD-34381, EMD-34382, EMD-34384, EMD-34383 and EMD-34385 respectively. Local refinements of the two Tt-SC IV2+I+III2+CII regions of Tt-MC (IV2+I+III2+CII)2 generated in this study have been deposited in the EMDB under accession codes EMD-34404 and EMD-34405 respectively.
The structural models used in this study are available in the PDB under accession codes 5XTI [https://www.rcsb.org/structure/5XTI], 1ZOY [https://www.rcsb.org/structure/1ZOY], 7W5Z [https://www.rcsb.org/structure/7W5Z], 7TGH [https://www.rcsb.org/structure/7TGH], 5IY5 [https://www.rcsb.org/structure/5IY5], 3CX5 [https://www.rcsb.org/structure/3CX5], 5J4Z [https://www.rcsb.org/structure/5J4Z], 6QBX [https://www.rcsb.org/structure/6QBX], 8BPX [https://www.rcsb.org/structure/8BPX].

Human research participants

Policy information about studies involving human research participants and Sex and Gender in Research.

Reporting on sex and gender	N/A
Population characteristics	N/A
Recruitment	N/A
Ethics oversight	N/A

Note that full information on the approval of the study protocol must also be provided in the manuscript.

Field-specific reporting

Please select the one below that is the best fit for your research. If you are not sure, read the appropriate sections before making your selection.

☒ Life sciences ☐ Behavioural & social sciences ☐ Ecological, evolutionary & environmental sciences

For a reference copy of the document with all sections, see nature.com/documents/nr-reporting-summary-flat.pdf

Life sciences study design

All studies must disclose on these points even when the disclosure is negative.

Sample size	Estimation of the sample size was mainly based on a previous study https://pubmed.ncbi.nlm.nih.gov/35357889/ and Cryo-EM studies of similar protein complexes. The numbers of micrographs and particles are presented in Supplementary Fig. 4, Supplementary Fig. 6 and Supplementary Table 1, which demonstrate that they are sufficient to generate map refinements at 2.80-3.26 Å for Tt-MC IV2+(I+III2+II)2 and 4.18-6.77 Å for Tt-MC (IV2+I+III2+II)2.
Data exclusions	There is no pre-set data exclusion criteria. Manual curation excluded less than 5% raw micrographs from both Datasets due to bad imaging quality or contamination. Particle curation by 2D classification and 3D ab-initio reconstruction were used to further exclude particles that would not contribute meaningfully to the final map reconstruction due to low signal-to-noise ratio or ice contamination. Details are described in Supplementary Fig. 4, Supplementary Fig. 6 and Methods.
Replication	Purification of Tetrahymena ETC megacomplexes were performed at least 7 times in LMNG and at least 5 times in digitonin, results form which agreed upon each other as shown in Supplementary Fig. 1. Different classification methods were employed to isolate particle subsets of Tt-MC IV2+(I+III2+II)2 and Tt-MC (IV2+I+III2+II)2, particle numbers from which were constant. 3D refinements of different kinds (homogenous, non-uniform, local refinements, with or without global and local refinements, with or without C2 symmetry) were performed for at least 66 and 43 times for Tt-MC IV2+(I+III2+II)2 and Tt-MC (IV2+I+III2+II)2 respectively, which gave reproducible maps in the same resolution range. Extinction coefficient and activity measurements were performed in triplicates with the same protein sample, where all attempts at replication were successful.
Randomization	Randomization was performed in resolution estimation based on Fourier Shell Correlation method (gold standard), where the dataset was randomly split into two halves and refined independently. This process is performed for each 3D refinement shown in Supplementary Fig. 4 and Supplementary Fig. 6. For purification experiments, different batches of Tetrahymena were cultured under identical conditions were considered equivalents. Randomization was therefore not necessary.
Blinding	Blinding was not performed to the studied sample since it is not a standard procedure in structural biology. Raw micrographs or particle images are not categorical data. Particles are randomly assigned into half-sets for image processing; hence no blinding is applicable. This study did not include experiments with experimental group allocation and thus no blinding was applied. All experimental samples were prepared and analyzed as described in the manuscript.

Reporting for specific materials, systems and methods

We require information from authors about some types of materials, experimental systems and methods used in many studies. Here, indicate whether each material, system or method listed is relevant to your study. If you are not sure if a list item applies to your research, read the appropriate section before selecting a response.

Materials & experimental systems

n/a	Involved in the study
☒	☐ Antibodies
☐	☒ Eukaryotic cell lines
☒	☐ Palaeontology and archaeology
☒	☐ Animals and other organisms
☒	☐ Clinical data
☒	☐ Dual use research of concern

Methods

n/a	Involved in the study
☒	☐ ChIP-seq
☒	☐ Flow cytometry
☒	☐ MRI-based neuroimaging

Eukaryotic cell lines

Policy information about cell lines and Sex and Gender in Research

Cell line source(s)	Tetrahymena thermoohila SB210
Authentication	Tetrahymena cell line was provided by the National Aquatic Biological Resource Centre, NABRC
Mycoplasma contamination	N/A
Commonly misidentified lines (See ICLAC register)	N/A
